# Monthly Summary

**Published:** 1896-01

**Authors:** 


### Monthly Summary.

PROF. MILLER OF BERLIN has proved that the active cause of dental caries is due to Bacteria in the mouth. A boon to dentists is BORINE the IDEAL DENTAL ANTISEPTIC. In its natural strength it will devitalize the mouth in a quarter of a minute while it does not harm the delicate epithelium or injure the dentine, a fact which cannot be claimed for any other antiseptic, as BORINE is not only a thorough germicide and prophylactic but is also *Non-Toxic and Non-Irritating*.

MEN THAT SHOULD NOT BE TOLERATED IN THE DENTAL PROFESSION.—The man who is never tired of repeating to everybody, and at all hours of the day, that he has been practicing dentistry for forty years.

The code-of-ethics man who believes that all advertising dentists should be hanged and quartered.

The paper-reading fiend that on every available occasion comes up with a paper bristling with scientific terms and with all the longest words we have in the English language, yet in substance says nothing that all his hearers haven't forgotten before they left the dental college.

The man who discusses such papers with the sole object of seeing his name in print.

The dental society men who mutually admire and soft-soap each other at every meeting or convention. I would suggest that these men found a dental mutual admiration society and relieve us from the suffering of having to read so much trash in the dental journals.

The tortuous nerve-canal perfect-filling crank.

The man whose papers have been written by somebody else and are produced as his own.

The man who cries down bridge-work because he doesn't know how to do it.

The man who claims that no dentist should undertake to perform any dental operation till he can do it in the most approved manner. This reminds me of the old woman who wouldn't allow her daughter to go bathing till she had learned how to swim.

The man who never makes a mistake, and who has never had a failure to record.—Francis Eschauzier, San Luis Potosi, Mexico, in *Southern Dental Journal.*

---

FILLING CHLDREN'S TEETH.—Children's teeth should be treated in a human manner. Gain their confidence if it requires a dozen visits before doing a thing for them. Begin early with their temporary teeth. Up to four years of age, these are not very sensitive. I do not agree with the essayist, when he says give them coarse foods to masticate. Their little teeth are not capable of crushing hard substances. While the calcification of the roots is incomplete, they are not strong enough to bear the pressure, and after absorption begins, the edges of the roots have jagged, sharp points. They can not eat such food, and when given i', alone, they can only bolt it whole; and dyspepsia follows. When the sixth year molars erupt, the roots are in the same incomplete state. Do not fill these teeth with gold. You will break the child down and fail to preserve the teeth. And do not use amalgam, as the teeth are soft and will be discolored by it. Use the cements and gutta-percha, replacing when necessary. You will, in this way make a life-long patient for yourself, or some one else. Use every effort to save the pulps till calcification has been completed. If the pulp is allowed to be devitalized from neglect, or from your applications, before the roots are formed, the apex being funnel shaped, with the enlargement the wrong way, your applications or your filling material will go through, and you may have necrosis or other serious trouble. A knowledge of physiology, histology and embryology is necessary for the dental operator, if he would avoid serious blunders.—Dr. T. M. Allen in *Items of Interest.*

---

VARNISHING CAVITIES.—Dr. W. G. Browne, of Atlanta, Ga., in *Items of Interest*, advocates the use of a clear resin, such as damson, dissolved in chloroform to line cavities before filling. It will act as a non-conductor of thermal changes, an insulator against electrical influences, will prevent discoloration from oxidization of amalgam, will help to support frail walls to a limited degree, and is useful in starting gold.

---

PULP-CAPPING IN DECIDUOUS TEETH.—In case of accidental exposure in excavating, fill a little saucer-shaped cup of platinum with a paste of oxid of zinc with carbolic acid and oil of cloves, equal parts, inverting over the point of exposure. Fill the cavity with oxiphosphate. The cup prevents pressure; the paste sterilizes the exposed point and allows healing by first intention.—T. G. Perry, in *Southern Dental Journal.*

